# Complete genome sequence of *Proteiniclasticum* sp. QWL-01, isolated from sewage sludge

**DOI:** 10.1128/MRA.00450-23

**Published:** 2023-08-03

**Authors:** Wanling Qiu, Junyu Zhu, Chao-Jen Shih, Yen-Chi Wu, Yi-Ting You, Chih-Hung Wu, Ching-Hua Liao, Shu-Jung Lai, Sheng-Chung Chen

**Affiliations:** 1 School of Resources and Chemical Engineering, Sanming University, Sanming City, Fujian, People’s Republic of China; 2 College of Environment and Safety Engineering, Fuzhou University, Fuzhou, Fujian, People’s Republic of China; 3 Bioresource Collection and Research Center, Food Industry Research and Development Institute, Hsinchu, Taiwan, Republic of China; 4 Department of Life Sciences, National Chung Hsing University, Taichung City, Taiwan, Republic of China; 5 Fujian Provincial Key Laboratory of Resources and Environmental Monitoring and Sustainable Management and Utilization, Sanming University, Sanming, Fujian, People’s Republic of China; 6 Graduate Institute of Biomedical Sciences, China Medical University, Taichung City, Taiwan, Republic of China; 7 Research Center for Cancer Biology, China Medical University, Taichung City, Taiwan, Republic of China; Queens College Department of Biology, Queens, New York, USA

**Keywords:** *Youngiibacter*, *Proteiniclasticum*, sewage sludge

## Abstract

Here, we report the complete genome sequence of *Proteiniclasticum* sp. QWL-01 (= BCRC 81396), isolated from sewage sludge of the Wastewater Treatment Plant of Sanming Steel Co. Ltd., Fujian, China. The genome of strain QWL-01 was selected for further species delineation and comparative genomic analysis.

## ANNOUNCEMENT

**Fig 1:**
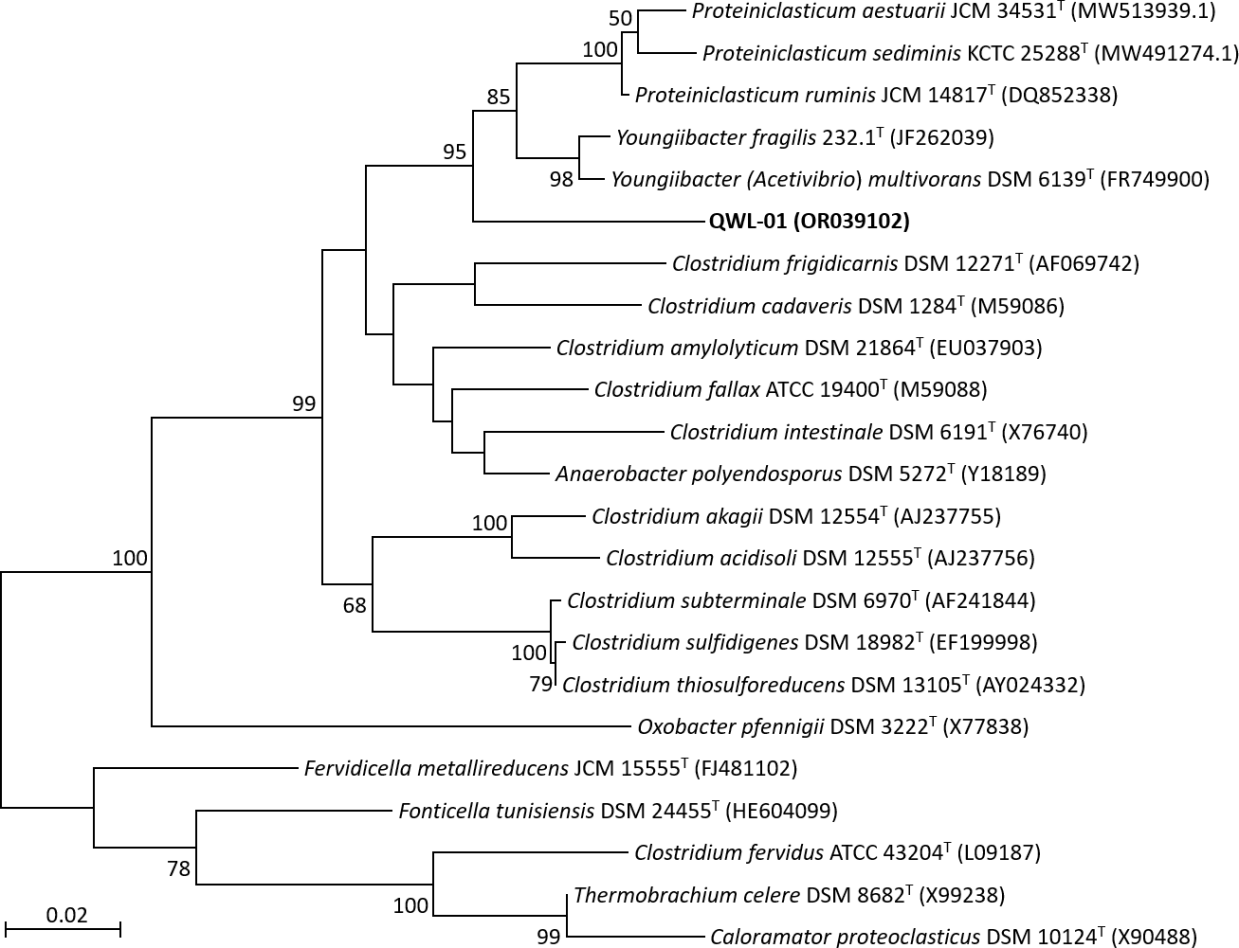
Maximum likelihood tree based on 16S rRNA gene sequences of strain QWL-01 and related taxa. Bar, 0.02 substitutions per nucleotide position. Bootstrap values were expressed as percentages of 1,000 replications.

Strain QWL-01 was isolated from sewage sludge of the Wastewater Treatment Plant of Sanming Steel Co. Ltd., Fujian, China. Sewage sludge was collected on 25 June 2021. The sample of sludge was inoculated into the anaerobic modified DSM 120 medium without the addition of acetate and methanol, prepared according to the instruction of the medium, and incubated at room temperature (~25°C) for 2 weeks. Strain QWL-01 was further purified and identified using the method combined with serial dilution, rolling-tube technique (1), and 16S rRNA gene clone sequencing with 8F (5′-AGAGTTTGATCCTGGCTCAG-3′) and 1492RU (5′-TTTTAATTAAGGTTACCTTGTTACGACTT-3′) primers (2) for three rounds. The purity of bacteria was verified by observation of morphology, 16S rRNA gene, and genome sequencing. Based on the analysis through EZBioCloud 16S rRNA gene database (3), strain QWL-01 showed highest similarities to both characterized and valid strains, *Youngiibacter multivorans* DSM 6139^T^ (4, 5) (93.82%) and *Proteiniclasticum ruminis* D3RC-2^T^ (6) (93.75%). Phylogenetic analysis of 16S rRNA gene sequences performed by MEGA11 (7) for strain QWL-01 and related taxa indicated that strain QWL-01 could be affiliated with a novel genus (Fig. 1). The genome of strain QWL-01 was selected to sequence for the species delineation and comparative genomic analysis.

The strain QWL-01 has been deposited in the Bioresource Collection and Research Center, Taiwan as strain BCRC 81396. It was grown in the modified DSM 120 medium and incubated at 30°C. The genomic DNA of strain QWL-01 was extracted using the NucleoBond HMW DNA Kit (Macherey-Nagel, Germany), according to the manufacturer’s instruction. The genome was sequenced at the Sangon Biotech (Shanghai) Co., Ltd. using the DNBSEQ-T7 platform (MGI Tech Co., Ltd.) and MinION sequencer (Oxford Nanopore Technology).

For the DNBSEQ-T7 platform, sheared genomic DNA fragments of approximately 300 bp were utilized to prepare a 150-bp paired-end DNA library. The library preparation was performed using Hieff NGS MaxUp II DNA Library Prep Kit for Illumina [Yeasen Biotechnology (Shanghai) Co., Ltd.]. The constructed library was sequenced using MGISEQ-2000RS High-throughput Sequencing Set (PE150 format), and 13,928,896 reads were generated and trimmed using Trimmomatic v0.36 (8). For MinION sequencer, a total of 542,078 reads were obtained, followed by trimming using Porechop v0.2.4 (9) and filtering using NanoFilt v2.8.0 (10). DNBSEQ-T7 and MinION reads were hybrid *de novo* assembled using Canu v2.2 (11). The hybrid sequencing protocol generated ~844× mean coverage of the genome. Gene predictions and annotations were performed using NCBI Prokaryotic Genome Annotation Pipeline v6.5 (12).

The assembly generated a single large contig of 3,691,162 bp with 50.81% GC content, which was circularized by aligning both ends of the contig sequences and deleting overlapped sequences of one end. The genome was predicted to have 3,360 genes, of which 3,232 were protein coding. The genome contains 15 rRNA genes and 53 tRNA genes. One clustered regularly interspaced short palindromic repeats with a high evidence level were found in the genome by using CRISPRCasFinder (13). Default parameters were used for all bioinformatics analyses.

## Data Availability

The genome sequence of strain QWL-01 has been deposited in GenBank under accession number CP120965. The version of the genome described in this paper is the first version. The BioProject and BioSample accession numbers are PRJNA949111 and SAMN33924797. DNBSEQ-T7 and MinION raw reads were deposited in the Sequence Read Archive (SRA) under accession numbers SRR24295755 and SRR24295756, respectively. The 16S rRNA gene sequence of strain QWL-01 has been deposited in GenBank under the accession number OR039102.
